# Role of Mitogen-Activated Protein Kinases in the Formation of Hypertrophic Scar with Model of Lipopolysaccharide Stimulated Skin Fibroblast Cells

**DOI:** 10.12669/pjms.341.13636

**Published:** 2018

**Authors:** Weidong Wang, Guanglei Li, Hongming Yang

**Affiliations:** 1 Dr. Weidong Wang, Chinese PLA Medical School, Beijing, China; 2 Dr. Guanglei Li, Chinese PLA Medical School, Beijing, China; 3 Dr. Hongming Yang, The First Affiliated Hospital of the Chinese PLA General Hospital, Fucheng Rd No. 51, Haidian District, Beijing, China

**Keywords:** Hypertrophic Scar, Fibroblast Cell, Lipopolysaccharide, Mitogen-Activated Protein Kinase

## Abstract

**Objective:**

Hypertrophic scar is common in burn patients, but treating result could not meet the expectation of the patients and doctors. We have found that certain concentration level of lipopolysaccharide (LPS) stimulated normal fibroblast cells have statistically similar with fibroblast cells from hypertrophic scar on the phenotype level, and with this work we are trying to figure out which Mitogen-Activated Protein Kinase (MAPK) is affected and how it is affected.

**Methods:**

Experiments were conducted in May, 2017 at the first affiliated hospital of the Chinese PLA General Hospital, Beijing, China. We have cultured the cell line of human skin fibroblast cells and randomly divided cells into four groups: control group and three stimulation groups. We have rebuilt the LPS stimulated model of skin fibroblast cells in hypertrophic scar based on our previous work. Experimental groups were stimulated with 0.1ug/mL LPS concentration for 24 hours, 48 hours, and 72 hours, respectively. Then we performed western blot analysis of Erk, p-Erk, JNK, p-JNK, p38 and p-p38. We performed statistical analysis with SPSS 15.0.

**Results:**

LPS can up regulate the MAPK/p38 pathway (p<0.05) and down regulate the MAPK/Erk and MAPK/JNK pathways (p<0.05). The changes of phosphorylated protein are time-related, with longer stimulation duration, significant difference is increased (p<0.05).

**Conclusion:**

MAPKs can play an important role in the formation of hypertrophic scar in the skin. Early intervention through the MAPKs could be a promising target in the prevention of the formation of hypertrophic scar.

## INTRODUCTION

The healing process helps patients restore the integrity of the skin, but the process could not be completed without scar. Depth of injury is a pivotal factor during the healing process in burn patients, because hypertrophic scar would format if the injury involves the dermis layer.^1^ Hypertrophic scar is one of the major problems in clinical practices. The unbearable itchy feelings, destruction of functions and appearance of the scar have caused great pain to the patients both physically and psychologically.

The formation of hypertrophic scar is a complex process and involves plenty of factors such as growth factors, proteolytic enzymes and extracellular matrix proteins. The matrix metalloproteinase-1,2,9 and tissue inhibitors of metalloproteinases were involved in the formation of hypertrophic scar, one study shows MMP-9 could play a leading role in scar-free healing.^2^ TGF-β isoforms are also involved in the process, studies shows that Smad pathways are believed to be the major pathway that regulate the formation of hypertrophic scar.^3^ Our previous study showed that gram-negative bacteria is responsible for the scar formation because many serious hypertrophic scars are formatted after serious infection in the wound skin area, and according to some studies infection is an important factor in the formation of hypertrophic scar.^4^-^6^ Further study by researchers shows that the immune response along with the severity of inflammation contributes to the formation of hypertrophic scar.^7^

Human skin fibroblast (HSF) plays an important role during the main reconstruction process of the damaged skin area. Researchers have found that fibroblast cells in hypertrophic scar behaved an altered phenotype than normal fibroblast cells.^8^ During our previous work, we have found that certain concentration level of lipopolysaccharide (LPS) stimulated normal fibroblast cells have statistically sameness with fibroblast cells from hypertrophic scar on the phenotype level.^9^-^11^ Now we are trying to use *in vitro* model to help understanding the changed behavior of the normal fibroblast cells, which factor would mainly lead to the change of normal fibroblast cells, and what is the mechanism beyond that.

Non-Smad signaling pathways have also been implicated with TGF-β signaling, but the exact mechanisms are not yet clear.^12^-^13^ The Mitogen-Activated Protein Kinase (MAPK) could conduct transmission of intracellular and extracellular signals, and could be stimulated by many components of the microenvironment. Binding of LPS to Toll-like receptors would affect their downstream signaling components including the MAPKs.^14^ Toll-like receptor 4 (TLR4) were located in skin fibroblast cells and periodontal fibroblast cells. And LPS was considered to be the stimulator of the TLR4 receptor.^15^ TLR-4 has a key function in defending against Gram-negative bacteria, viruses, fungi and mycoplasma. There are three major subfamilies in the MAPK: extracellular signal-regulated kinase (Erk), p38 and c-Jun-N terminal kinase (JNK).^16^

The MAPK/Erk pathway is a major regulator in varies cellular processes including cell proliferation, differentiation, adhesion, migration and survival. The MAPK/JNK pathway could be stimulated by cytokines, growth factor deprivation and G protein coupled receptors and stress signaling. The MAPK/p38 signaling pathway could be influenced by varies stress stimulations including heat, osmotic shock and inflammatory cytokines.^17^

LPS is known to be the stimulator of MAPKs. The phosphorylation of Erk, JNK and p38 could be up regulated when facing the stimulation of LPS.^18^ But how MAPKs regulate during the LPS stimulation in skin fibroblast cells needs to be investigated. Previous work of PL. Wang *et al* in the periodontal fibroblast cells showed that TLR4 and the activation of MAPK/JNK and MAPK/p38 were involved.^19^ Yet little has been studied on the skin fibroblast cells. Thus the question arises that when LPS stimulates normal skin fibroblast cells into hypertrophic scar ones on the phenotype level, whether MAPKs are involved and which pathway is involved needs to be further looked into. We have tried to answer this question in this study.

## METHODS

Experiments were conducted in May, 2017 at the first affiliated hospital of the Chinese PLA General Hospital, Beijing, China. High-Glucose Dulbecco's Modified Eagle's Medium (DMEM-H) was obtained from Hyclone (South Logan, UT), Fetal Bovine Serum (FBS) was obtained from Gibco (Waltham, MA). Complete medium formula was 94% DMEM-H and 6% FBS. Antibodies were obtained from Cell Signaling Technology (Danvers, MA) unless otherwise stated: p38 MAPK antibody, phospho-p38 MAPK antibody, JNK1/2/3 antibody (Huaxingbio, Beijing, China), phospho-SAPK/JNK antibody, p44/42 MAPK antibody, phospho-p44/42 MAPK antibody, β-actin antibody, anti-rabbit IgG, HRP-linked antibody. Lipopolysaccharides (LPS) was obtained from Sigma (Ontario, CA) and was diluted into 1000ug/mL with H_2_O, then the solution was diluted into 1ug/mL and 0.1ug/mL respectively with complete medium. D-Hank's solution was obtained from Solarbio (Beijing, China). SDS-PAGE Sample Loading Buffer was obtained from Beyotime (Jiangsu, China).

### Cell culture and stimulation

Cell line of Human Foreskin Fibroblast (HFF) were obtained from the China Center for Type Culture Collection (CCTCC). Cells were cultured in 100mm plates with 10ml complete medium under the condition of 37°C and 5% CO2 in a humidified incubator. Stimulation of LPS was conducted as follows: discard 1mL complete medium in the plates and replace it with 1mL LPS (1ug/mL) solution that would make the concentration of the LPS 0.1ug/mL in the plates, non-stimulation groups were replaced with 1mL complete medium at the same time. When came to the second and third stimulation time point, the post-stimulated plates were replaced with 0.1ug/mL LPS solution which would still make the concentration of the LPS 0.1ug/mL in all the stimulated plates. Stimulation was proceeded on 72 hours, 48 hours and 24 hours before harvest the cells.

### Protein preparation

We practiced all the following steps on ice. Cells are washed with cold (4°C) D-Hank's solutions for three times, then were harvested by adding cold (4°C) RIPA lysis buffer (150 mM NaCl, 1% NP-40, 0.5% deoxycholic acid, 0.1% SDS, 50 mM Tris pH=8.0) with protease inhibitor and phosphotransferase inhibitor to plates followed by scraping. Harvested cells were placed on ice for 30 minutes with vortex shaker for 20 seconds every 10 minutes. Then centrifuge the samples at 12000g at 4°C for 30 minutes and transfer the supernatant into clean tubes. SDS-PAGE Sample Loading Buffer were added to the samples followed by boiling at 95°C for five minutes and then storaged at -80°C until protein detection by Western blot analysis.

### Western blot analysis

Electrophoresis was performed with lysate protein samples loaded on a 10% SDS-PAGE gel. We transfered the protein samples onto a nitrocellulose membrane and blocked with 5% nonfat milk (to detect β-actin and non-phosphorylated proteins) or 5% BSA solution (to detect phosphorylated proteins) for one hour. After blocking, we incubated the membrane with primary antibodies with the dilution ratio of 1:1000 overnight at 4°C on the shaker. On the next day, we washed the membranes for three times five minutes each followed by incubation with the secondary antibodies with the dilution ratio of 1:2000 for one hour. Then we washed the membranes again with previous washing method, and used GE Image Quant LAS 4000 system (GE, Boston, MA) to visualize the protein bands. Data for semi quantitative analysis were obtained with Gel-pro (Media Cybernetics, Rockville, MD)

### Statistical analysis

We have conducted all experiments for at least three times. Statistical analysis was performed with the SPSS 15.0 software. The data was evaluated using Student's t-test, a value of p<0.05 was considered as statistically significant.

## RESULTS

**Fig. 1:**
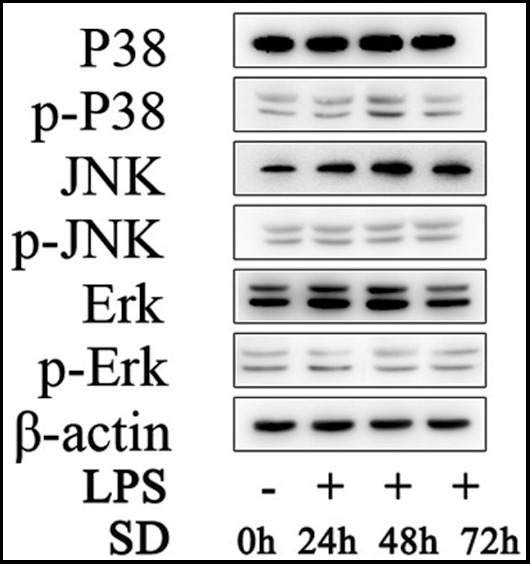
Protein bands of western blotting.

**Fig. 2:**
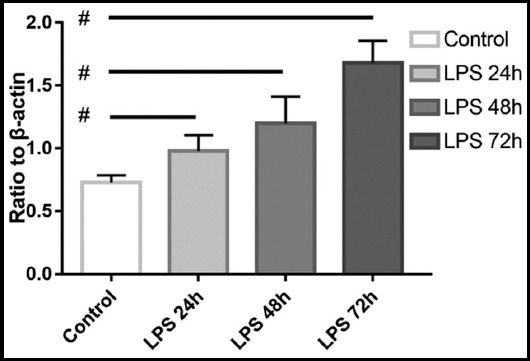
Ratio of p-Erk against β-actin The p value comparing the experimental group and control group are 0.027, 0.035, 0.019, respectively. Symbol # indicate p>=0.05 compared with control group.

**Fig. 3:**
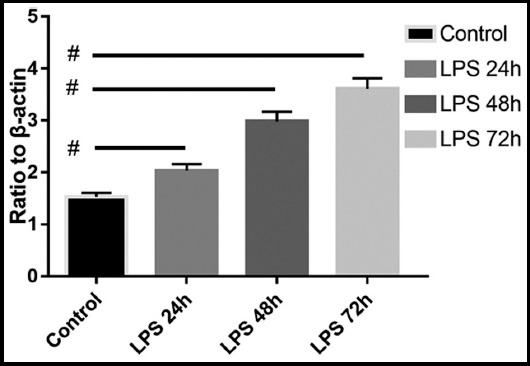
Ratio of p-JNK against β-actin The p value comparing the experimental group and control group are 0.014, 0.002, 0.006, respectively. Symbol # indicate p>=0.05 compared with control group.

**Fig. 4:**
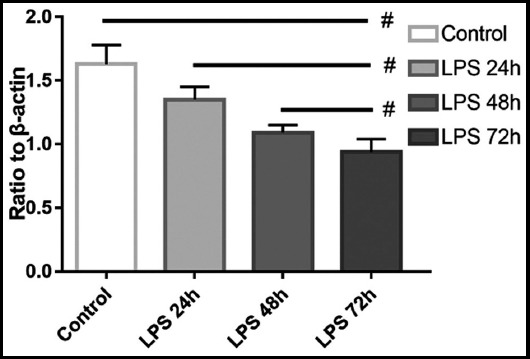
Ratio of p-p38 against β-actin The p value comparing the experimental group and control group are 0.021, 0.048, 0.042, respectively. Symbol # indicate p>=0.05 compared with control group.

The protein expression levels of Erk, p-Erk, JNK, p-JNK, p38 and p-p38 were determined by western blot and quantification analysis. β-actin served as a loading control during western blot. The visualized protein bands are shown in Fig.1. Ratio of target protein against β-actin are shown in Table-I and phosphorylated proteins are shown in Fig. 2-4. The LPS (0.1ug/mL) stimulation duration (SD) of fibroblast cells are 0 hour (Control), 24 hours (LPS 24h), 48 hours (LPS 48h) and 72 hours (LPS 72h). The values are mean ± SD of three independent experiments.

**Table-I:** Results of semi quantitative data analysis.

Target Protein	Control	LPS 24h	LPS 48h	LPS 72h
Erk	0.64±0.06	0.657±0.07*	0.70±0.09*	0.74±0.08*
p-Erk	0.73±0.06	0.98±0.13#	1.20±0.21#	1.68±0.18#
JNK	0.45±0.06	0.53±0.07*	0.68±0.08*	0.83±0.11*
p-JNK	1.53±0.08	2.03±0.13#	2.98±0.19#	3.16±0.20#
P38	1.05±0.09	0.93±0.08*	0.88±0.13*	0.66±0.10*
p-p38	1.63±0.15	1.35±0.10#	1.09±0.06#	0.94±0.10#

Note: Listed data are summary of three independent experiments; original data are ratios of target protein to β-actin. Symbol # indicate p>=0.05 compared with control group, and * indicate p<0.05.

The results of stimulating skin fibroblast cells indicates that the phosphorylation levels of Erk and JNK are up regulated when stimulated by certain concentration level (0.1ug/mL) of LPS (p<0.05 of all groups), the phosphorylation level of p38 is down regulated when stimulated by the LPS (p<0.05 of all groups). The changes of phosphorylated protein are time-related, with longer stimulation duration, comes more significant differences (p<0.05). Levels of total Erk, JNK and p38 comes without statistical difference when compared to the control group (p>0.05).

## DISCUSSION

In this study, we are aiming in MAPKs changes in human skin fibroblast cells with LPS stimulation model. Our experimental results demonstrate that LPS inhibits the MAPK/p38 pathway and stimulates the MAPK/Erk and MAPK/JNK pathways *in vitro* in Human Skin Fibroblast cells. In addition, the LPS stimulation could manipulate the phosphorylation of the MAPKs by up regulating the p-Erk, p-JNK and down regulating the p-p38. All data shows a time-dependent manner.

We have reported previously that the CD14 and TLR4 are involved in the proliferation of LPS stimulated normal fibroblast cells and concluded that these receptors could have an important role in the formation of the hypertrophic scar.^20^,^21^ In addition, this study could move one-step further in better understanding the formation of the hypertrophic scar.

Currently, the treatment of hypertrophic scar is limited to surgery, radiotherapy and other non-surgical therapy,^22^ thus after-healing treatment. Nevertheless, the therapeutic effect remains far from satisfaction because surgical procedure takes a long time to recover and the outcome fall short of expectation. Better understanding of the mechanism underlying the formation of hypertrophic scar may help to develop new strategy for improving the therapeutic efficacy of treating hypertrophic scar.

The MAPKs are one of the important factors that play a critical role in the inflammatory processes. Many studies have showed that the phosphorylation levels of Erk, JNK and p38 were increased when stimulated with LPS. While different cell types could respond dissimilarly to the LPS, our results suggest that same type of cells, fibroblast cells, also could be different. Compared with the work of PL. Wang *et al* of gingiva fibroblast cells that with LPS stimulation, they observed an up regulation of MAPK/JNK and MAPK/p38 pathways^23^, yet our study suggests the different way with skin fibroblast cells.

Some studies^24^,^25^ suggest that the wound in the oral mucosa heals much faster and formats less scar than the wound in the skin. If the gingiva fibroblast cells function like the skin fibroblast cells in scar formation, what makes the difference in them? Could cell transplantation of the gingiva fibroblast cells into the wound area on the skin get less hypertrophic scar? Or in a rather simple way, how to manipulating the MAPKs in a way that could lead a better outcome? For our future work, we could continue focusing on the downstream of MAPKs in skin fibroblast cells, after better understanding of MAPKs.

### Limitations of the study.

The formation of hypertrophic scar is a complex process that involves many other factors. The role of MAPKs is only a tip of an iceberg, although we have observed the effects of LPS can change the MAPKs to up or down regulation, how the detailed mechanisms of molecules still needs further investigation. Furthermore, after applying the inhibitor of activated pathways, how the fibroblast cells' proliferation, apoptosis and differentiation were affected after the LPS stimulation, whether *in vitro* findings in this study are applicable for *in vivo* remains to be addressed.

## CONCLUSION

Along with our previous work results that LPS were involved in the progression of stimulating normal skin fibroblast cells into hypertrophic scar ones, combined with the results of this study, the MAPKs could play as pivotal intracellular signal transducers in skin fibroblast cells. The cells' changing of phenotype from normal ones into hypertrophic scar ones is very much likely through up regulating the MAPK/p38 pathway and down regulating the MAPK/Erk and MAPK/JNK pathways. This means that MAPKs could play an important pathophysiological role in the formation of the hypertrophic scar. Early intervention through the MAPKs could be a promising target in the prevention of the formation of hypertrophic scar.

### Authors' Contribution

***WW:*** Designed the experiment, did data collection and manuscript writing.

***GL:*** Did statistical analysis and editing the manuscript.

***HY:*** Did review and final approval of the manuscript.
